# Transversely pumped laser driven particle accelerator

**DOI:** 10.1038/s41467-026-72697-x

**Published:** 2026-05-02

**Authors:** Tanner Nutting, Nicholas Ernst, Alexander G. R. Thomas, Karl Krushelnick

**Affiliations:** 1 Gérard Mourou Center for Ultrafast Optical Science, University of Michigan, Ann Arbor, MI USA; 2 Department of Nuclear Engineering and Radiological Sciences, University of Michigan, Ann Arbor, MI USA

**Keywords:** Plasma-based accelerators, Laser-produced plasmas, Plasma physics

## Abstract

We present a novel acceleration scheme capable of accelerating electrons and ions in an underdense plasma. Transversely Pumped Acceleration (TPA) uses multiple arrays of counter-propagating laser beamlets that focus onto a central acceleration axis. Tuning the injection timing and the spacing between the adjacent beamlets allows for precise control over the position and velocity of the intersection point of the counter-propagating beam arrays. This results in an accelerating structure that propagates orthogonal to the direction of laser propagation. We present the theory that sets the injection timing of the incoming pulses to accelerate electrons and ions with a tunable phase velocity plasma wave. Simulation results are also presented which demonstrate 1.12 GeV proton beams accelerated in 3.6 mm of plasma and electron acceleration gradients on the order of 1 TeV/m in a scheme that circumvents dephasing. This work has potential applications as a compact accelerator for medical physics and high energy physics colliders.

## Abstract (web summary)

Spatiotemporally structured laser fields are attracting interest for laser-based particle acceleration schemes. Here, the authors introduce transversely pumped acceleration, demonstrating potential practical advantage over previously established schemes and opening towards compact accelerators for applications.

## Introduction

The concept of using plasma waves produced by lasers to accelerate electrons in an underdense plasma^1^ has matured into a well-developed field with impressive accomplishments such as experimental observations of electron beams on the order of 10 GeV^2,3^ in recent years. Spatiotemporal control of a laser’s focal position and velocity^4^ has been proposed as a method to go beyond limits to acceleration in laser-driven particle accelerators. Advanced focusing concepts offering spatiotemporal control of a laser’s focal position and velocity^5–7^ have been proposed for a variety of applications^8–10^.

Laser wakefield acceleration (LWFA) is fundamentally limited by dephasing: as electrons gain energy, they outrun the accelerating phase of the plasma wave, preventing further energy gain. Traditional LWFA schemes are therefore constrained by the dephasing length, *L*_*d*_, which scales as

$${L}_{d} \sim \frac{{\uplambda}_{p}^{3}}{{\uplambda }_{0}^{2}}\propto {n}^{-3/2},$$

where *λ*_*p*_ is the plasma wavelength, *λ*_0_ the laser wavelength, and *n* the plasma density. Overcoming this limit is critical for increasing electron energies without requiring prohibitively long acceleration distances or complex staging.

Recent efforts have explored new regimes for dephasingless acceleration by leveraging the aforementioned spatiotemporally structured laser fields. Debus et al.^11^ demonstrated that the phase velocities of an accelerating structure can be controlled using pulse front tilted laser pulses in plasma. This allows electrons to remain in the accelerating phase of the wake without needing to tailor the density profile of the plasma^6^. Caizergues et al.^8^ and Palastro et al.^9^ pioneered the use of the luminal wakefield and flying focus concepts in LWFA, where the intensity peak of a focused, radially delayed laser pulse travels at a controllable velocity by means of a stepped echelon and an axiparabola^12–14^. This allows the plasma wake to be continuously driven at a phase velocity that is matched to that of the electrons.

The axiparabola is a reflective optic that produces an extended focal line. This optic has emerged as a key tool for extending the interaction length between laser fields and plasmas while maintaining relativistic intensities^12,13^. Recent developments have expanded the utility of the axiparabola by combining it with spatiotemporal coupling techniques, such as the previously mentioned flying-focus configurations and tailored pulse-front velocities, to control the trajectory and velocity of the laser’s peak intensity^14,15^. These capabilities open new possibilities for synchronizing laser intensity with plasma wave dynamics, mitigating dephasing, and enabling novel acceleration geometries. Experimental and simulation studies have confirmed the robustness of axiparabola-focused pulses^16^. In this work, we introduce an novel approach to spatiotemporal structuring of light for the acceleration of charged particles.

An application of particular interest for high-energy physics^17^ involves the use of such spatiotemporally controlled lasers for electron acceleration. Dephasingless schemes allow for operation at higher plasma densities, enabling a larger accelerating field (since the accelerating fields scale as *E* ∝ *n*^1/2^, where *n* is the plasma density). This has been investigated in simulations using pulse front tilt^11^ and stepped echelons^9^ for electron acceleration.

An additional application of spatiotemporally structured light in underdense plasmas is the acceleration of ions, which could be performed by matching the phase velocity of the plasma wave to the velocity of the accelerating ion bunch. Simulations of this concept have been demonstrated by the use of a chirped pulse with a diffraction grating and a lens such that the focal position of the laser moves transversely to the propagation of the laser^10^.

In this work, we present the concept of Transversely Pumped Acceleration (TPA), a versatile accelerator concept that allows for customized tuning of plasma wave phase velocity. The concept of transversely pumped laser acceleration naturally invites the use of arrays of high repetition rate laser beams and the use of efficient optimization techniques and machine learning. We demonstrate applications of this proof of principle concept using the OSIRIS Particle in Cell (PIC) code. We show in this work that electrons and ions can be accelerated in this scheme. This work has potential applications in compact proton therapy and positron acceleration as well.

**Fig. 1:**
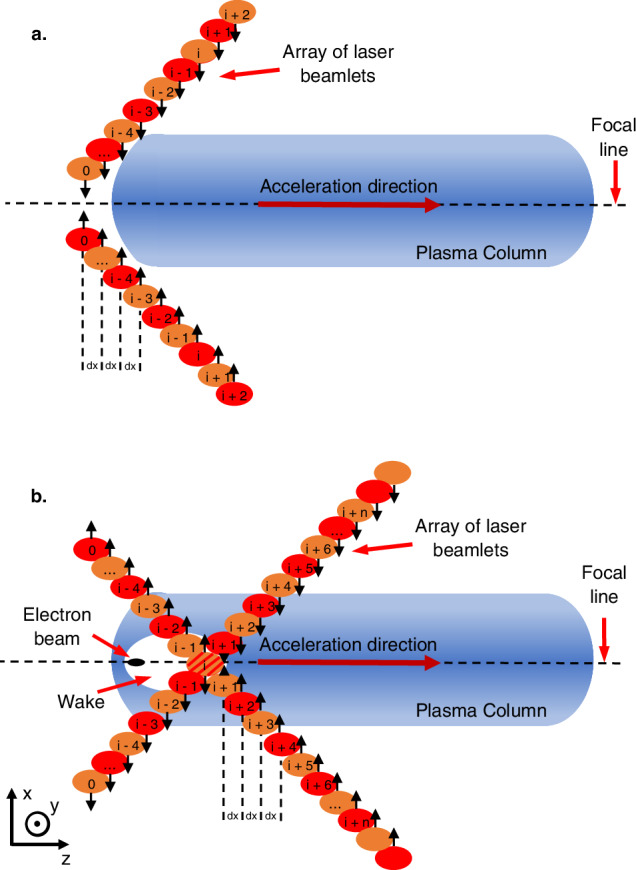
Schematic of transversely pumped acceleration scheme. **a** TPA concept prior to laser beamlets entering plasma. Red and orange beamlets, separated by distance *dx*, represent different laser polarizations. Each pair of incoming pulses is labeled in relation to *i* and is focused onto the focal line. Black arrows show the direction of propagation of each beamlet. The laser propagation direction is transverse to the particle acceleration direction. **b** TPA concept at a later time. Beamlet arrays crossing at the focal line produce a wake and can result in the injection of an electron beam.

TPA uses multiple arrays of counter-propagating, focused laser beamlets. The adjacent pulses within each laser beamlet array are delayed with respect to one another such that the intersection point of the incoming beamlet arrays on the focal line propagates at a desired velocity. A schematic of the proposed acceleration concept is shown in Fig. 1. Multiple laser beamlet arrays are injected symmetrically onto a line-focus in the center of the plasma column. The direction of particle acceleration is transverse to the direction of laser beamlet propagation. Laser propagation directions are denoted with black arrows. The top illustration in Fig. 1 shows the scheme prior to the crossing of the beamlet arrays and the bottom illustration shows the scheme after the beamlet arrays cross and a wake has been produced in the plasma column.

Coherence effects from the incoming lasers in TPA can cause laser beating, which negatively affects the stability of the accelerating structure for both ion and electron acceleration. TPA can use alternating cross-polarized beamlets to avoid these effects. Figure 1 illustrates a schematic of the TPA concept in the context of electron acceleration. Arrays of laser pulses, consisting of individual beamlets, propagate orthogonal to the acceleration direction, as shown by the arrows. The arrays of laser beamlets cross at the focal line, which is shared by each array of pulses. The timing and spacing between the adjacent pulses can be adjusted to give an arbitrary and variable phase velocity of the accelerating structure, such as *c*.

TPA offers attractive advantages over existing schemes for dephasingless electron acceleration as well as ion acceleration. Existing dephasingless electron acceleration concepts are limited when scaling up to obtain electron energies in the TeV range. Using stepped echelons to impart radial pulse delay onto a laser pulse to be focused by an axiparabola^9^ is limited by the amount of radial pulse delay that can be imparted. At this electron energy scale, one must operate the accelerator over several meters, requiring on the order of 10 ns of radial pulse delay. Another scheme proposes the crossing of two counter-propagating laser pulses with pulse front tilts^11^. This concept is limited by the amount of energy that can be put into the two laser pulses, requiring large and expensive optics to reach high energies. Although the accelerating structure of the scheme we propose here is related to the scheme of Debus^11^, the ideas are different in their fundamental limitations and advantages. The primary advantages of the newly introduced TPA scheme are the flexibility and the scalability of the scheme. This scalability is an essential advantage in the field of dephasingless acceleration since one can utilize the high acceleration gradients for as long as is desired.

TPA is not limited by the aforementioned factors and is also useful for ions. Additionally, TPA is unlimited in terms of its length scalability and, therefore, electron energy gain. In this scheme, it is only necessary to add more moderate energy laser pulses to the arrays of laser beamlets to achieve more acceleration. An additional advantage of using many moderate to low-energy laser pulses in the TPA scheme is the ability to operate at a higher repetition rate. This is desirable for industrial and medical applications, and enables the use of machine learning to optimize parameters. The use of many low-energy laser beamlets also allows for the use of smaller optics, significantly reducing cost. In addition, it is possible to create a plasma wave of arbitrary phase velocity using this scheme.

The advantages of TPA are also applicable to ion acceleration in an underdense plasma. The energy gain of the scheme in ref. ^10^ is limited by the pulse length of the chirped laser pulse. For longer pulses, more energy needs to be added to the laser to maintain the same peak intensity. Additionally, the scheme in ref. ^10^ requires complicated optics that may be difficult to manufacture, whereas TPA’s most complicated aspects are alignment and timing, both of which can be controlled electronically.

TPA’s transverse geometry also allows for flexibility in the injection of beams into the accelerating structure. In this work, we simulate a proton beam that could be produced by Radiation Pressure Acceleration (RPA)^18^ and inject it into the accelerating structure (see the section titled Ion Acceleration Simulations for more details). Note that the proton beam to be accelerated could also be directly produced in the plasma.

## Results

### Phase matching

In practice, controlling the phase velocity of the plasma wave requires control over the injection timing of each set of laser pulses, with the goal of producing a structure that propagates with a phase velocity equal to the velocity of the particles being accelerated. Consider a coordinate system with $\widehat{{{\bf{z}}}}$ being the acceleration direction. A train of pulses, each labeled *i*, propagate in the direction **k** with ${{\bf{k}}}\cdot \widehat{{{\bf{z}}}}={{\bf{0}}}$. This pulse train is timed to make a controllable intensity maximum on axis. The position of the intensity maximum is *s*(*t*) with the resulting intensity pulse designed to move with an axial group velocity function *V* = *ds*/*dt* and with axial intensity profile *I*(*ζ*, *t*), where *ζ* = *z* − *s*(*t*).

For a particle accelerated along the *z*-axis starting at *z* = 0 in a wake with electric field, *E*_*z*_(*z*), from the equation of motion the differential of the particle momentum is *dp*_*z*_ = (*qE*_*z*_/*v*_*z*_)*dz*. Hence, we may write

1 $${s}^{-1}(z)={\int }_{\!\!\!\!0}^{{p}_{z}(z)}\frac{{v}_{z}}{V}\frac{d{p}_{z}^{{\prime} }}{q{E}_{z}}\,.$$

Under phase matching conditions with *V*(*z*) = *v*_*z*_, and with the time averaged driver being constant (see Supplementary Fig. 1 for details), such that the electric field is on time averaged constant *E*_*z*_ = E^*^, then

2 $${s}^{-1}(z)=\frac{{p}_{z}(z)-{p}_{0}}{q{E}^{*}}\,,$$

where *p*_0_ is the initial particle momentum and the *z* dependent momentum ${p}_{z}(z)=mc\sqrt{{\gamma }^{2}-1}$ can be found by integrating the equation for the particle kinetic energy, *γ*(*z*)*mc*^2^ = *γ*_0_*mc*^2^ + qE^*^z. Hence,

3 $${t}_{i}=\frac{mc}{q{E}^{*}}\left(\sqrt{{\left({\gamma }_{0}+\frac{q{E}^{*}{z}_{i}}{m{c}^{2}}\right)}^{2}-1}-\frac{{p}_{0}}{mc}\right)\,,$$

Consider two cases of interest:

- Ultrarelativistic electron, ${\left({\gamma }_{0}+\frac{q{E}^{*}{z}_{i}}{m{c}^{2}}\right)}^{2}\gg 1$ and $\frac{{p}_{0}}{mc}\simeq {\gamma }_{0}$,
  4 $${t}_{i}\simeq \frac{{z}_{i}}{c}\,,$$
  i.e., the group velocity is constant at the speed of light *c*.
- Proton accelerated from rest, *γ*_0_ = 1 and *p*_0_ = 0, then
  5 $${t}_{i}=\frac{{z}_{i}}{c}\sqrt{1+\frac{2m{c}^{2}}{q{E}^{*}{z}_{i}}}\,.$$
  In the case that qE^*^z_*i*_/*mc*^2^ ≪ 1, i.e., the particle remains non-relativistic, then
  6 $${t}_{i}\simeq \sqrt{\frac{2m{z}_{i}}{q{E}^{*}}}\,.$$

In the following sections, we demonstrate these concepts in particle-in-cell simulations.

### Ion acceleration simulations

A structure suitable for the acceleration of heavier, positively charged particles, such as protons, has been developed and tested using the theoretical tools developed in the previous section. For this study, consider a quasi-monoenergetic proton beam with peak energy at 95 MeV and a full width at half maximum (FWHM) energy spread of 20 MeV, reasonably produced by an RPA solid target source^19^. Protons with an energy of 95 MeV have a velocity of approximately 0.42*c*, and can be injected into the TPA accelerating structure. It is critical to match the initial phase velocity of the accelerating plasma wave to the initial velocity of the injected ion beam by inserting the correct value of *γ*_0_ into Eq. (3).

**Fig. 2:**
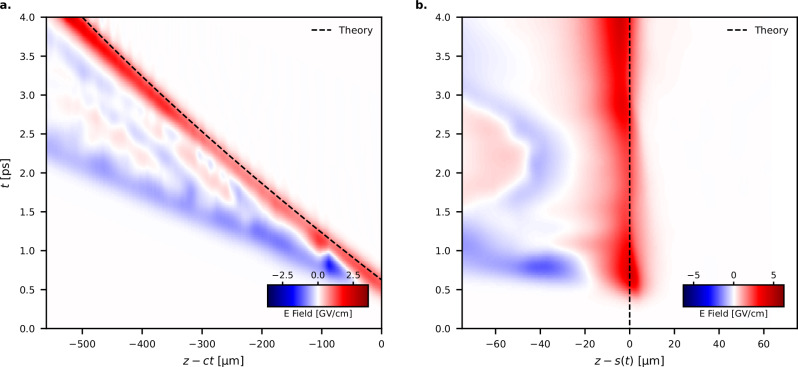
Temporal evolution of on-axis accelerating field for ion acceleration. **a** Streak plot of the on axis accelerating fields for ions in the speed of light frame. **b** Streak plot of the same accelerating fields in the frame co-moving with the ion beam that is being accelerated. The theoretical predictions for both streak plots are shown as a dashed black line.

To illustrate that the accelerating structure produced in simulations matches the theory of the previous section, two streak plots of the on axis accelerating fields produced by the plasma wave are shown in Fig. 2. Both plots are of the same simulation, but are presented in different frames of reference. Figure 2a illustrates the temporal evolution of the accelerating fields in the speed of light frame. The accelerating structure starts slower than the speed of light (0.42*c*) and accelerates as the simulation progresses. As one may expect, the accelerating region slips back in the speed of light frame, and follows a curve with a positive second derivative due to the acceleration of the structure. If run longer, the curve traced out by the accelerating region would asymptotically approach a vertical line as the speed of the structure nears *c*.

Figure 2b illustrates the accelerating field of the structure in the frame co-moving with the ion beam that is being accelerated. In agreement with the theory, the accelerating region traces a vertical line. This indicates that the position of the accelerating region is nearly constant in the frame *z* − *s*(*t*).

**Fig. 3:**
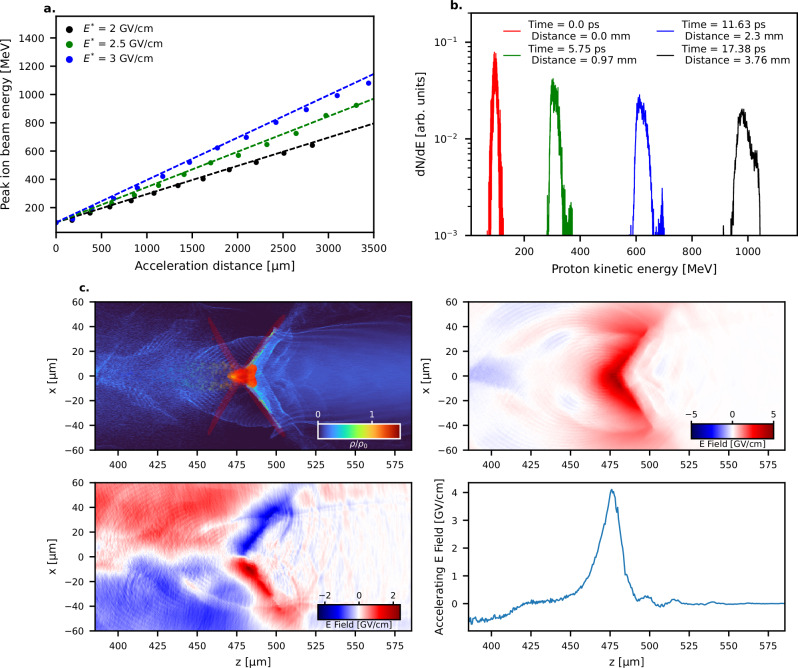
Proton acceleration results and accelerating structure. **a** Peak ion energy as a function of acceleration distance for different values of E^*^. A maximum proton energy of 1.12 GeV was obtained in 3.6 mm for E^*^ = 2.5 GV/cm. **b** Ion energy spectrum at different times for E^*^ = 2.5 GV/cm. **c** (Top left) Ion beam in red, shown in accelerating structure. The electron density is shown in blue and the laser pulses are shown as red contours. (Top Right) Accelerating field structure. (Bottom left) Focusing field structure. (Bottom right) Line-out of accelerating field structure along the central axis of the simulation.

Figure 3c illustrates an example of the accelerating structure for ions. An ion beam, shown in red in the top left figure, is accelerated along the leading edge of the crossing point between the incoming laser beamlets. For the parameters shown in these simulations, the accelerating ions are near the intersection point of the laser beamlet arrays, meaning they could be subjected to pondermotive effects over a few millimeters of acceleration. This may affect beam properties such as emittance and divergence and could be studied more closely in future work.

This accelerating structure is highly tunable. The initial velocity and the acceleration of the structure is set by the transverse spacings of the laser beamlets and the temporal delays between the pulses. The strength of the accelerating field depends both on the plasma density and the laser beamlet intensity. This is because a higher laser intensity results in the displacement of more electrons and therefore, a higher electric field near the crossing point of the beamlets.

With an accelerating structure that ensures phase matching between the accelerating ion beam and the velocity of the plasma wave, the acceleration of protons to relativistic energies can also be demonstrated. The injected ion beam has a Gaussian density profile in both dimensions with FWHM = 4 μm and has a density of 1.5 × 10^16^cm^−3^. If cylindrical symmetry were assumed for this beam, the resulting charge would be on the order of ~ 10 pC.

The accelerating structure was produced in an underdense hydrogen plasma with density 1.8 × 10^18^cm^−3^ and 290 mJ laser pulses. Additional information on the pulse parameters for these simulations can be found in the Methods section. 1200 beamlets were used per array to accelerate protons over a distance 1200 × *dx* = 3.6 mm, where *dx* = 3 μm. Adjacent laser pulses were cross-polarized with respect to one another, polarizations are in $\widehat{z}$ and $\widehat{y}$ directions.

A plot showing the proton energy gain vs. acceleration length for the simulation parameters described above for various values of E^*^ is shown in Fig. 3a. The dashed lines on the plot indicate theoretical predictions. Each simulation used the same plasma and laser parameters. The maximum ion energy obtained for a value of E^*^ = 2.5 GV/cm was 1.12 GeV, with a peak energy of 0.98 GeV, demonstrated after 3.6 mm of acceleration. The quasi-monoenergetic nature of the initial ion beam is preserved during acceleration in the TPA scheme. Figure 3b shows the ion spectrum at 4 different times during the acceleration process for a value of E^*^ = 2.5 GV/cm. The energy spread of the initial injected ion beam is 20 MeV FWHM, while the final energy spread is 40 MeV FWHM. This result demonstrates the stability of the structure and the effectiveness of the phase matching over several millimeters. Similar structures could in principle also be used for positron acceleration.

### Dephasingless electron acceleration simulations

TPA’s versatility enables its use for dephasingless electron acceleration as well. This is possible due to the ability to set an arbitrary phase velocity of the plasma wave. By setting the velocity of the accelerating structure equal to *c*, dephasingless operation is achieved. This allows for the use of higher-density plasmas, therefore increasing acceleration gradients.

**Fig. 4:**
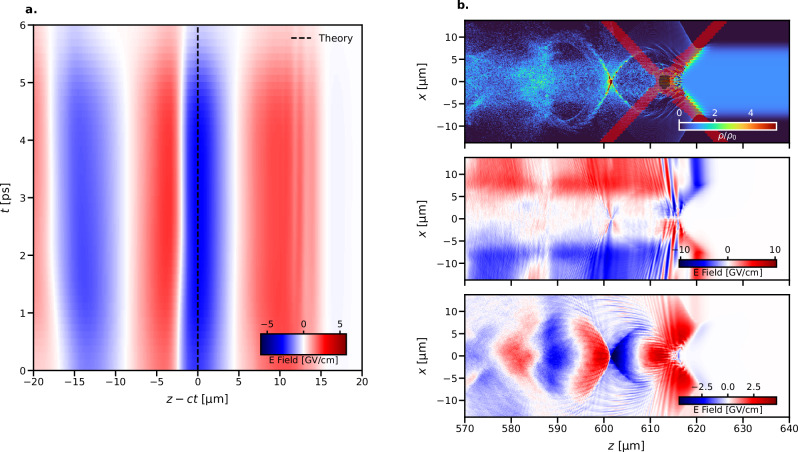
Dephasingless electron acceleration structure and temporal evolution. **a** Streak plot showing a phase velocity of *c*. The on axis accelerating fields are shown as a function of time and space. The black dashed line denotes a perturbation with a velocity of *c*, showing agreement of simulation with theory. **b** (Top) Electron density of accelerating structure. Laser contours are shown in red. The energy per laser pulse is 7 mJ. (Middle) Focusing fields. (Bottom) Accelerating fields.

In a similar fashion to that done for the ion acceleration case, the phase velocity of the accelerating structure is confirmed to be *c* through a streak plot shown in Fig. 4a. Here, the on axis accelerating fields are plotted in the speed of light frame. The region where electrons are accelerated appears stationary in the speed of light frame, implying electrons being accelerated in the wake will never dephase. The vertical dashed line shows the theoretical prediction of the location of the accelerating structure in both time and space.

The accelerating structure in the case of electron acceleration is shown in Fig. 4b. The plots in this figure are produced with adjacent laser beamlets that are in phase with one another and polarized in the $\widehat{y}$ direction. This was done for the purpose of illustrating the focusing and accelerating fields. A practical implementation of the concept would use cross-polarized adjacent beamlets since this eliminates the need to initialize adjacent pulses in phase with one another.

This structure was produced in a plasma with density *ρ* = 1.2 × 10^18^cm^−3^ (or 0.012*n*_*cr*_) using laser pulses with energy equal to 7 mJ, readily produced at high repetition rate. Using higher-density plasmas will produce higher accelerating gradients while still avoiding dephasing. However, the intensity of each laser beamlet must be increased for higher-density operation in order to produce the accelerating structure. For this study we chose to operate at *ρ* = 1.2 × 10^18^cm^−3^ since this density was high enough to demonstrate the out-performance of a traditional laser wakefield accelerator in terms of avoiding dephasing. Yet, the chosen density was low enough to demonstrate what modest energy laser beamlets (7 mJ) are capable of producing in the TPA arrangement. Additional information regarding the electron beams produced in this configuration are shown in Supplementary Fig. 3.

The electrons are injected into the structure via self-injection during the collapse of the plasma column back into itself in the wake of the crossing laser beamlet arrays. The temporal evolution of the injection is shown in Supplementary Fig. 2. In practice, ionization injection, shock injection, or even injection using a separate linac could be used.

### Three-dimensional simulation results

**Fig. 5:**
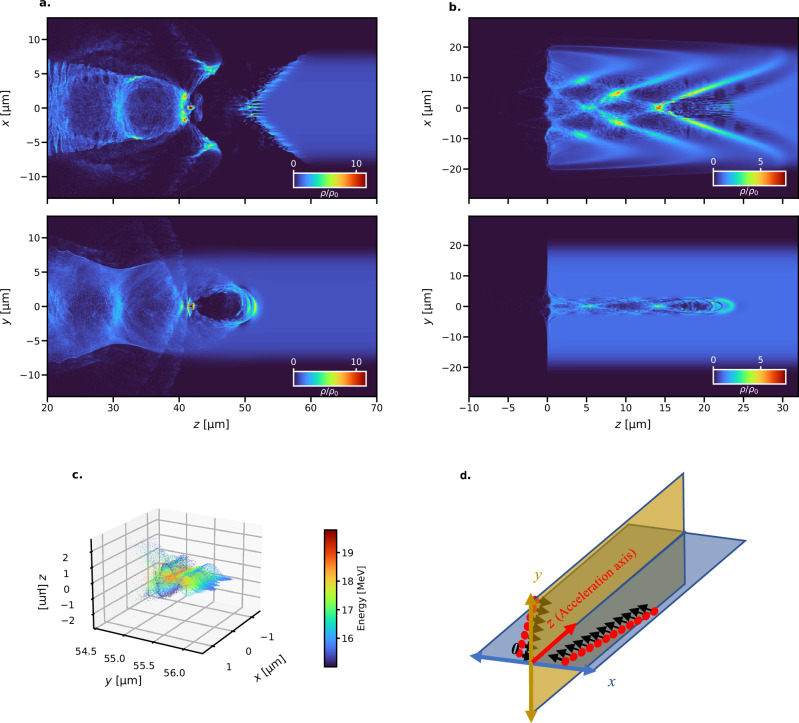
Three-dimensional simulation results. **a**, **b** Cross sections of the electron density profiles for 3D simulations demonstrating the accelerating structure in a 16(40) μm FWHM plasma column. The top plots show the cross-section of the *y* = 0 plane, while the bottom plots show the cross-section of the *x* = 0 plane. **c** Scatter plot of 3D electron beam. Energies of particles are given in the color bar. Note that this scatter plot shows electrons with energies > 15 MeV. **d** Geometry of the 3D simulations in this study.

Proof of principle simulations were conducted for the case of electron acceleration in three dimensions using OSIRIS. The conducted study examined the case of using two beamlet arrays, but this concept could use more beamlet arrays coming in from different angles with respect to the acceleration axis. Because of computational cost limitations, we only simulated these structures for short distances. These beamlet arrays were injected into the simulation box in the same plane (*y* = 0) and came to focus along a central axis, were *x* = *y* = 0. The individual beamlets traveled in the $\pm \widehat{x}$ direction and crossed at *x* = 0, where the beams came to focus, and the accelerating structure was produced. See Fig. 5d for a visualization of the geometry.

Two cases are presented here. The first of which uses a plasma column width of 16 μm FWHM with density *ρ* = 1.2 × 10^18^cm^−3^ and laser beamlet energies *E* = 7 mJ. The accelerating structure formed in the electron density profile is shown in Fig. 5a. The top and bottom plots are slices through the center of the plasma profile. The top slice shows the plane in which the lasers are traveling. The lasers are traveling from top to bottom and vice versa in the top slice. The lasers are traveling in and out of the page in the bottom slice. Note that the top slice is the *y* = 0 plane and the bottom slice is the *x* = 0 plane.

The maximum energy in the electron beam produced in this simulation is 20 MeV after an acceleration distance of 50 μm. This corresponds to an acceleration gradient of 0.4 TeV/m. The acceleration length for this study was limited by computational feasibility. However, the structure of the wake and temporal evolution appears similar to that of the two-dimensional simulations. It is likely that optimization of parameters in the 3D case could improve the ultimate energy spread of the accelerated beam.

A 3D plot of the electron beam is presented in Fig. 5c for energies > 15 MeV. The charge of this beam is calculated to be 0.78 pC for electron energies > 15 MeV and 0.93 pC for electron energies > 12.5 MeV. The efficiency, i.e., the total energy in the electron beam divided by total laser energy used, was calculated to be 3 × 10^−5^ for the 3D case. This is an example simulation and is not optimized and could be improved. The beam is slightly elongated in the $\widehat{x}$ direction as a result of the asymmetric wake. This demonstrates the production of tailorable electron beam shapes. Using only two beamlet arrays results in an asymmetric wake and electron beam. The addition of more beamlet arrays coming in from additional angles could bring more symmetry to the structure. Consequently, this highlights the flexibly of the scheme. The number of beamlet arrays used can shape the accelerating structure and electron beams. For example, flat (asymmetric-emittence) beams are of interest in the linear collider community^20^. It is reasonable to assert that changing the configuration of the incoming beamlets could allow for custom wake shapes, leading to unique electron beam properties.

The second three-dimensional simulation presented in this work demonstrates that the accelerating structure does not need to occupy the entire width of the plasma column. This case, shown in Fig. 5b, uses a plasma column width of 40 μm FWHM with the same density as the previous case and pulse energies *E* = 830 μJ. The wake in the second case is composed of a plasma bubble that does not take up the entire width of the plasma column.

Note that using a plasma filament would reduce any refractive effects from plasma ionization. The beamlets would only propagate through tens of microns of plasma. Additionally, any refractive effects could be accounted for prior to the beamlets entering the plasma since the discrete nature of the idea allows for such fine-tuning. It is also possible to preionize the plasma.

The inconsistencies in the accelerating structure between the three-dimensional and two-dimensional cases are primarily due to a fundamental difference in the geometry of the simulation. The two-dimensional case mimics an infinite plane of the interaction, which differs from that of a standard three-dimensional case. Ideally, the three-dimensional case would be explored further via a proof of principle experiment, rather than further numerical simulations, due to significant computation load and cost.

## Discussion

TPA is a novel acceleration scheme that offers many advantages over existing acceleration concepts. Accelerating ions in an underdense plasma circumvents the need for high contrast laser pulses needed for solid target experiments^21^. Using multiple moderate intensity laser pulses in tandem with a gas target could also allow for higher repetition rate ion acceleration, something that is presently difficult for solid target experiments. Time-domain multiplexing techniques^22^ provide a means of obtaining intensities/energies needed in the TPA laser pulses.

Traditionally, the three primary limitations to laser-driven electron acceleration are dephasing, depletion, and defocusing, all of which are bypassed using TPA. Results demonstrated in this work show TPA could allow for the production of a TeV electron beam in 2.5 m with the potential to be shortened if operated at a higher plasma density. The laser energy required for 1 TeV electrons is estimated to be 27 kJ using 2 million beamlets with energies of 7 mJ each. The number of beamlets used can be reduced by using more intense lasers and a higher-density plasma or by using beamlets with larger spot sizes. For example, we have run simulations using 10 J per pulse in a 10^19^electrons/cm^3^ plasma and observed 2.5 TeV/m acceleration gradients with spacings of 3 μm between adjacent beamlets. This requires 160,000 beams and 1.6 MJ to reach 1 TeV electron beams in 40 cm. It is important to state that these numbers are not fixed and can change depending on things like available laser energy, focusing, or desired acceleration gradient.

**Fig. 6:**
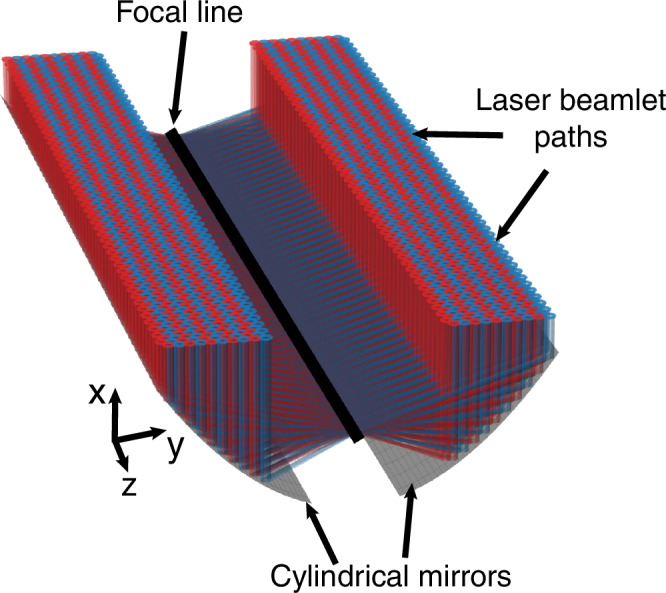
Schematic of practical implementation of TPA concept. Implementation of TPA with staggered beamlet arrays in the near field, each delayed with respect to its neighbor to achieve a programmable velocity at focus. Beamlet arrays are focused using two cylindrical optics. Overlap of the pulses at focus is achieved through staggering beamlets in the near-field. Note that other angles could be used to enable transmission of unused laser energy.

A potential three-dimensional rendering of the TPA concept is shown in Fig. 6. Arrays of laser beamlets, such as those that could be produced for the near field of a fiber laser array are focused using cylindrical mirrors. The relative timing between the beamlets could be modified with sub-femtosecond control^23^. The overlap of the pulses at the focus can be achieved by staggering the transverse positions of the beamlets on the cylindrical optic. This results in a continuous perturbation at focus.

This approach draws parallels to coherent fiber combining^23^, where the temporal spacing between the pulses could be adjusted with a Delay and Phase Adjustment Module. Additionally, we note that in ref. ^24^, fiber laser pulses are combined in phase with one another (i.e., sub-femtosecond control), showing temporal synchronization of many laser pulses is possible. The temporal synchronization requirements for TPA are less stringent since the plasma response is driven by a ponderomotive effect. The temporal spacings between the pulses are 4.3 and 10 fs for the electron and ion simulations shown in this work. This temporal spacing depends on the spacing between the pulses, which could be increased with larger spot sizes.

Fiber laser pulses are inherently much more efficient than other sources and as such, would have a wall plug efficiency of up to 25%^25^. Since the geometry is transverse, collection of the spent beamlets would be possible after the interaction with the plasma, the energy from these spent pulses could be recycled.

Perhaps the most unique and important advantage of TPA is the simplicity of scaling the accelerating structure to longer accelerating distances; scaling only requires additional laser beamlets and a longer cylindrical focusing optic. There is no need for wavefront shaping of a short pulse or precise chirping of a longer laser pulse, both of which would require specialized optics that may prove difficult to produce. TPA offers a straightforward method of tuning plasma wave phase velocity with applications in ion and electron acceleration. An additional advantage of the scheme is that the use of low-power lasers could allow for high repetition rate corrections to electron beam parameters by adjusting laser beamlet parameters and timings. Electron beam quality could be tailored to a given application dynamically based on figures of merit set by the user.

Instead of using arrays of fibers, a second method could involve the use of high-power laser systems, which have large (30 cm) beams. These beams could be masked, using apodizers, splitting the beam into a one or two-dimensional array of beamlets that could then be focused with independent angles into the plasma. Changing the delay between these beamlets would require changing the path lengths between the mirrors on a ~ 10 μm scale length, which is straightforward in the lab.

This scheme doesn’t necessarily need to use thousands of beams. We chose to use so many beams to demonstrate what is possible with low-energy (7 mJ) beamlets. These low-energy beamlets require a tight focus to reach a sufficient intensity. If larger spot sizes were used, less beams would be required, and the spacing and pointing fluctuations requirements would be less stringent. Note also that machine learning techniques have been employed to reduce root mean square pointing error to 0.34 μrad^26^.

The use of low-energy pulses is important in that higher repetition rate operation will be possible. Free-flowing gas targets can be used at high repetition rate to compliment the claimed high repetition rate operation enabled by the use of low-energy laser pulses^27^. Things like alignment can be automated with machine learning methods, making this scheme advantageous in aspects such as scalability and versatility. Machine learning and optimization methods are now widely used in plasma accelerator experiments^28^.

TPA could allow for an increase in average power of laser-generated particle sources while simultaneously making them more compact. Low-energy lasers can operate at a much higher repetition rate and average power than high-energy systems. Therefore, it is necessary to combine many lower-energy pulses to increase flux while maintaining beam quality.

The practical realization of TPA reduces to three requirements: (i) generating multiple delayed beamlets whose overlap region translates along a common focal line at a prescribed velocity, (ii) reaching relativistic intensity while remaining below optical-damage fluence limits, and (iii) implementing an experimental geometry that does not require an impractical number of independently aligned fast optics. These requirements are compatible with near-term laser technology because the essential task is not to surround the plasma with many independently stabilized small *f*/*#* beamlines, but to transport energy through a large aperture and synthesize a moving overlap region on a shared focal line. In the continuous limit, this corresponds to a rectangular beam incident on a cylindrical (line-focusing) optic, where a controlled transverse delay in the near field produces an apparent motion of the peak intensity along the focal line. A discrete implementation replaces the continuous beam by an array of beamlets, each delayed relative to its neighbor but focused onto the same line; a schematic is shown in Fig. 6.

Overlap at focus does not require near-field overlap: beamlets can be generated and delayed upstream (e.g., via fiber or segmented architectures), then astigmatically line-focused so that they overlap only at the interaction line. For example, overlap in the propagation direction can be obtained using a near-field matrix in which adjacent beamlets are transversely offset by one-half of the beamlet diameter. Coherent fiber arrays have demonstrated residual inter-channel phase errors as low as *λ*/90 RMS^23^, which at *λ* = 1 μm corresponds to an equivalent timing error

7 $$\delta t \sim \frac{\uplambda /90}{c}\approx 0.037\,{{\rm{fs}}},$$

well below the multi-femtosecond inter-beamlet spacings considered here. This architecture directly addresses optical crowding near the target: the dominant scaling constraint is optical fluence (single-aperture transport), rather than a requirement for many independently stabilized free-space beamlines.

A simple scaling example makes this explicit. Using the manuscript parameters (*dx* = 1.3 μm) over an interaction length *L* = 2.6 m implies

8 $$N \sim \frac{L}{dx} \approx 2 \times {10}^{6}\,\,\,{{\rm{beamlets}}} \; {{\rm{per}}} \; {{\rm{side}}}\,.$$

With beamlet energies ~ 7 mJ, the required energy is ~ 15 kJ per side. Assuming a conservative damage-limited fluence *F* ≲ 1 J/cm^2^, the required transverse clear-aperture dimension *d* for transporting energy *E* over length *L* scales as

9 $$d \, \gtrsim \frac{E}{F\,L}\approx \frac{1.5\times 1{0}^{4}\,{{\rm{J}}}}{(1\,{{\rm{J}}}/{{{\rm{cm}}}}^{2})(260\,{{\rm{cm}}})}\approx 58\,{{\rm{cm}}},$$

corresponding to an optic area of order (2.6 m) × (0.6 m). While large, this is fundamentally a single-aperture scaling problem, analogous to other large-aperture high-energy laser transport challenges.

An internal consistency check shows that such apertures can simultaneously satisfy intensity requirements. Focusing energy *E* into a line-focus region of transverse width *w* and length *L* with pulse duration *τ* gives

10 $$I \sim \frac{E}{w\,L\,\tau }.$$

Taking *w* ~ 3 μm, *L* = 2.6 m, *τ* ~ 20 fs, and *E* ~ 15 kJ yields *I* ~ 10^19^ W/cm^2^, i.e., squarely in the relativistic regime required for wake excitation.

From a practicality standpoint, the most compelling validation path is staged experimental demonstration:

1. Proof-of-principle wake excitation: demonstrate that two opposing beamlet arrays, line-focused with cylindrical optics, drive a wake whose excitation follows the programmed delay pattern.
2. Velocity control: vary imposed delays and directly observe the corresponding change in effective wake phase velocity.
3. Scaling: increase beamlet number and reduce spot size only after the control principle is experimentally validated.

This emphasizes that feasibility is established by validating the core control mechanism first, rather than immediately attempting the most aggressive parameter set. Proof of principle experiments could be conducted with apodized beams (such as from the ZEUS laser facility^29–31^) and a pulse front tilt.

To illustrate feasibility with energies and powers available at near-future high-energy ultrashort-pulse infrastructure, consider an NSF OPAL^32,33^ class pulse with *E* ~ 500 J and *τ* ~ 20 fs delivered in a line-focus geometry. If a symmetric configuration shares the relativistic intensity requirement between two opposing beams (targeting *I* ~ 5 × 10^18^ W/cm^2^ per beam) and the transverse line width is *w* ~ 3 μm, then the interaction length sustained at fixed intensity is

11 $$x=\frac{E}{I\,w\,\tau }\approx 17\,{{\rm{cm}}}.$$

With gradients discussed in this work (e.g.,  ~ 0.4 TeV/m), this corresponds to ~ 60–70 GeV energy gain in a single stage. For such facilities, the required transverse delay can be implemented via pulse-front tilt in a scheme conceptually similar to Debus^11^, but instead using apodized beams. A conservative fluence constraint *F* ~ 1 J/cm^2^ over *x* ≈ 17 cm implies a transverse transport dimension *d* ~ *E*/(*Fx*) ≈ 30 cm; for moderate *f*/*#* ~ 5, this corresponds to meter-scale focal lengths that are realistic for upcoming large-aperture beamlines.

## Methods

PIC simulations were performed using the OSIRIS 4.0 framework. A hydrogen plasma was used in each simulation with an 8th-order super-gaussian profile in the transverse directions. Each laser beamlet in the discussed simulations was initialized individually in OSIRIS input files that were produced using an automated text file generator to control laser parameters such as spacings and temporal delays between adjacent pulses. Both two-dimensional and three-dimensional simulations were ran for electron acceleration. Only two-dimensional simulations were ran for the ion acceleration case due to the large simulation box sizes needed for ion acceleration.

### Ion acceleration

The ion acceleration simulations in Fig. 3a, b used a 3.76 mm × 169 μm simulation box and ran for 17.38 ps. The accelerating structure was programmed to start at 0.42*c* and accelerate according to the predetermined E^*^ factor. A proton beam with an initial velocity equal to 0.42*c* was timed to inject into this accelerating structure. The laser pulses used to produced this structure were cross-polarized from their adjacent and intersecting pulses. Each beamlet of the incoming arrays had a normalized vector potential *a*_0_ = 8, beam waist *w*_0_ = 3 μm, pulse duration *τ* = 25 fs, and spacing *dx* = 3 μm. These parameters correspond to laser pulses with 290 mJ of laser energy per pulse.

The ion acceleration simulations in Figs. 2 and 3c used a 682 × 253 μm simulation box and ran for 4.1 ps. The laser pulses used in these simulations were all polarized in the $\widehat{y}$ direction and were initialized in phase with one another so that the accelerating and focusing fields could be clearly visualized. All ion acceleration simulations were ran in a static window due to the accelerating velocity of the region of interest.

### Electron acceleration

The two-dimensional electron acceleration simulations, such as those in Fig. 4, were ran using a moving window with size 92.4 × 27.5 μm and ran for 6.17 ps. As with all of the simulations presented in this work, the laser pulses were injected from the wall and propagated in directions perpendicular to the acceleration direction. Appropriate parameters for electron acceleration using the TPA concept are as follows: spacing between adjacent beamlets *dx* = 1.3 μm, beam waist of laser *w*_0_ = 1.64 μm, pulse duration *τ* = 20 fs, normalized vector potential *a*_0_ = 2.5, laser wavelength *λ* = 1 μm and plasma density *ρ* = 1.2 × 10^18^cm^−3^ (or 0.012*n*_*cr*_).

The three-dimensional electron acceleration simulation shown in Fig. 5a, c was ran using a 92.4 × 26.9 × 26.9 μm static window and ran for 280 fs. The parameters for the 16 μm FWHM plasma profile simulation are as follows: spacing between adjacent beamlets *dx* = 1.3 μm, beam waist of laser *w*_0_ = 1.64 μm, pulse duration *τ* = 20 fs, pulse energy *E* = 7 mJ, laser wavelength *λ* = 1 μm and plasma density *ρ* = 1.2 × 10^18^cm^−3^ (or 0.012*n*_*cr*_). These parameters correspond to laser pulses with normalized vector potentials equal to 2.5.

The simulation in Fig. 5b was run using a 50.4 × 60.5 × 60.5 μm static window and ran for 280 fs. The parameters for this case are as follows: plasma column width  = 40 μm FWHM, spacing between adjacent beamlets *dx* = 1.3 μm, beam waist of lasers *w*_0_ = 1.64 μm, pulse duration *τ* = 20 fs, pulse energy *E* = 830 μJ, laser wavelength *λ* = 1 μm, and plasma density *ρ* = 1.2 × 10^18^cm^−3^ (or 0.012*n*_*cr*_). These parameters correspond to laser pulses with normalized vector potentials equal to 0.85.

## Supplementary information

Supplementary Information

Transparent Peer Review file

## Data Availability

The raw simulation outputs generated during this study comprise approximately 4 TB of data and are not publicly available due to their size, which exceeds typical repository storage limits. The input files and processed datasets underlying all figures are available from the corresponding author upon request.

## Code availability

The simulations were performed using a modified version of the OSIRIS 4.0 framework to enable exceptionally long input configurations required for this study. The modified source code is available from the corresponding author upon request.
