# Supplementary material for: Transversely pumped laser driven particle accelerator
Source: Nat Commun. 2026 May 2;17:5949. doi: 10.1038/s41467-026-72697-x (PMC13341759; doi:10.1038/s41467-026-72697-x)
Supplement: Supplementary file 1 — Supplementary Information [file 41467_2026_72697_MOESM1_ESM.pdf]

Supplemental information: Transversely pumped laser driven particle accelerator

Tanner Nutting, Nicholas Ernst, Alexander G. R. Thomas, and Karl Krushelnick
Gérard Mourou Center for Ultrafast Optical Science, University of Michigan,
2200 Bonisteel Boulevard, Ann Arbor, Michigan 48109, USA
(Dated: April 13, 2026)

I. CORRECTION OF LASER INTENSITY WITH VARYING TEMPORAL SPACING BETWEEN PULSES

In practice, controlling the phase velocity of the plasma wave requires control over the injection timing of each set of laser pulses. Consider a coordinate system with $\hat{\mathbf{z}}$ being the propagation direction. A train of pulses, each labeled i, propagating in the direction $\hat{\mathbf{k}}$ with $\hat{\mathbf{k}} \cdot \hat{\mathbf{z}} = 0$ is timed to make a controllable intensity maximum on axis. The position of the intensity maximum is $s(t)$ with the resulting intensity pulse designed to move with an axial group velocity function $V = ds/dt$ and with axial intensity profile $I(\zeta, t)$, where $\zeta = z - s(t)$. The pulses are assumed to have a slowly varying complex envelope $A_i(\mathbf{x}, t)$, $||(\nabla A_i)/A_i|| \ll k_i$ and $||(\partial_t A_i)/A_i|| \ll \omega_i$, such that the vector potential $\mathbf{A}(\mathbf{x}, t)$ of the summed combinations of the pulses may be described by

$$\mathbf{A}(\mathbf{x}, t) = \Re \sum_i \hat{\mathbf{e}}_i A_i e^{i(\mathbf{k}_i \cdot \mathbf{x} - \omega_i t)} \; , \tag{1}$$

where $\mathbf{k}_i$, ω_i are the (central) wavevector and frequency of each pulse and $\hat{\mathbf{e}}_i$ is a vector describing the polarization, which is assumed to be a constant and normalized $|\hat{\mathbf{e}}_i|^2 = 1$. The ponderomotive force depends on $\langle \mathbf{A}^2 \rangle$, where the angle-brackets refer to a time average over fast oscillations. Therefore,

$$\langle \mathbf{A}^2 \rangle = \left\langle \frac{1}{4} \sum_{i,j} (\hat{\mathbf{e}}_i \cdot \hat{\mathbf{e}}_j) A_i A_j e^{i(\mathbf{k}_i + \mathbf{k}_j) \cdot \mathbf{x} - i(\omega_i + \omega_j) t} + (\hat{\mathbf{e}}_i \cdot \hat{\mathbf{e}}_j^*) A_i A_j^* e^{i(\mathbf{k}_i - \mathbf{k}_j) \cdot \mathbf{x} - i(\omega_i - \omega_j) t} + c.c. \right\rangle . \tag{2}$$

Considering that the only contributions come from states where A_i and A_j overlap in space/time, by alternating the polarization between orthogonal states with sufficiently spatiotemporally spaced pulses, as in this work, or in general by choosing different frequencies for the individual pulses so that the time average $\langle e^{i(\omega_i - \omega_j)t} \rangle$ is zero except for $i = j$, this reduces to the *incoherent sum*,

$$\langle \mathbf{A}^2 \rangle = \frac{1}{2} \sum_i |A_i(\mathbf{x}, t)|^2 \; . \tag{3}$$

In general, the full spatio-temporal profile of the resulting intensity profile can be derived. For simplicity, here we only consider the formation of the intensity maximum on axis (at $\mathbf{x}_\perp = \mathbf{0}$ for a single train of pulses and its dependence on distance propagated, and assume that the transverse distribution is symmetric, with pulses entering simultaneously from a number of angles, N_θ, about the z axis. We further assume that each pulse has an identical form, described by a single function $g(z, t) \in [0, 1]$, with each pulse focused on axis at a position along the z-axis, z_i, and arriving at $\mathbf{x}_\perp = \mathbf{0}$ at time t_i such that $A_i(\mathbf{x}_\perp = \mathbf{0}, z, t)/\sqrt{2} = a_i g(z - z_i, t - t_i)$, where a_i is some relative amplitude of each pulse, or

$$\langle \mathbf{A}^2 \rangle (\mathbf{x}_\perp = \mathbf{0}, z, t) = \sum_i a_i^2 |g(z - z_i, t - t_i)|^2 \; . \tag{4}$$

Setting $z_i = s(t_i)$ such that $\zeta = z - s(t_i)$ at $t = t_i$, which means that the axial intensity profile of the controllable intensity maximum is simply the transverse profile of each pulse in the z-direction, and using the properties of the Dirac delta distribution $\delta(x)$,

$$U_P(\zeta, t) = \langle \mathbf{A}^2 \rangle (\mathbf{x}_\perp = \mathbf{0}, z, t) = \int |g(\zeta, t')|^2 \rho(t - t') dt' \; , \tag{5}$$

where $\rho(t) = \sum_i a_i^2 \delta(t - t_i)$, i.e., the convolution of the function $g(\zeta, t)$ with a (non-uniformly spaced) comb of δ-distributions with amplitude a_i^2.

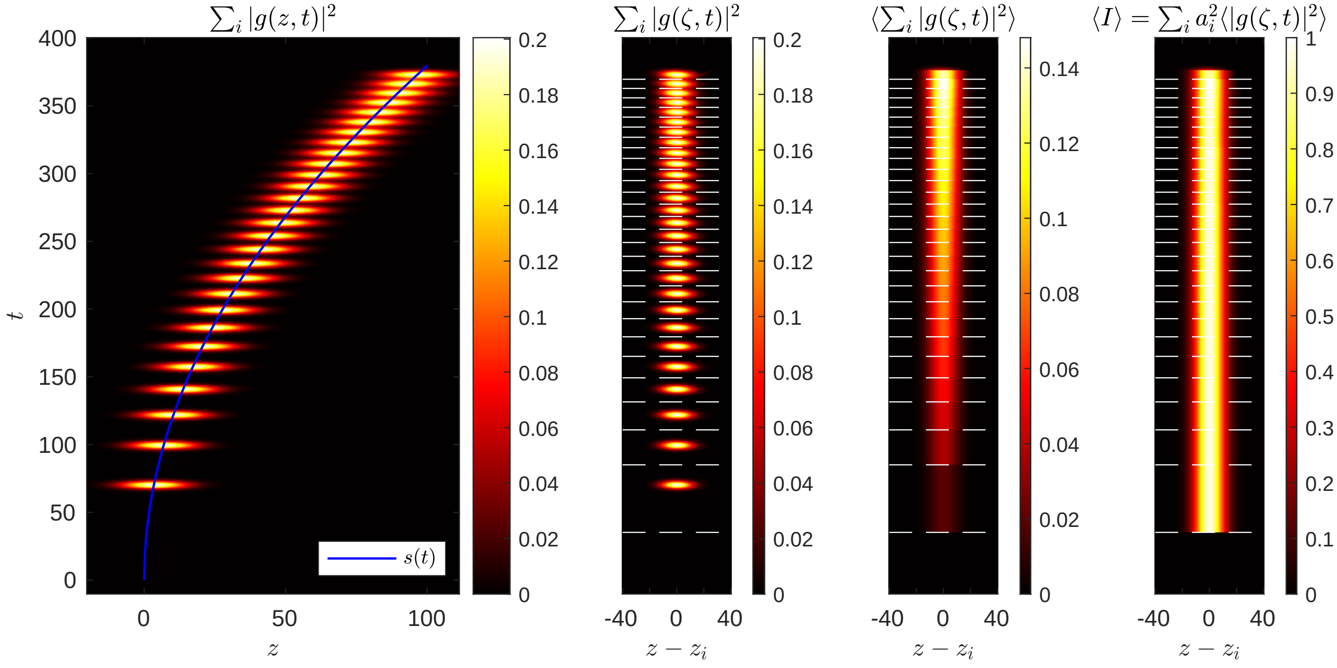

Supplementary Figure 1. Time averaging of pulse train. The colormap images show an example pulse train or time averaged pulse train as indicated in each subpanel title. The blue line indicates the desired trajectory $s(t)$. The white dashed lines show the intervals for time averaging as described in the text. The final panel shows that a weighted intensity for each pulse leads to a constant time-averaged intensity peak.

Here, we chose the spacing of the pulses in z to be uniform, $z_{i+1} - z_i = \Delta z =$ constant. This means that we may write the time delay in terms of the inverse function of $s(t)$ as $t_i = s^{-1}(z_i)$, with $s(t)$ defined above. However, this means that, in general, the temporal spacing between the pulses, t_i, is non-uniform. To see this, we may take the time average of $I(\zeta, t)$ over an interval T,

$$\langle U_P(\zeta, t) \rangle = \frac{1}{T(t)} \int_{T(t)} U_P(\zeta, t') dt' \ , \tag{6}$$

where $\int_{T(t)}$ indicates the integral is taken over an interval of width T containing t and allowed to vary in width with t. Hence,

$$\begin{aligned} \langle U_P(\zeta, t) \rangle &= \int |g(\zeta, t')|^2 \left[\frac{1}{T(t)} \int_{T(t)} \rho(t'' - t') dt'' \right] dt' \\ &\equiv \int |g(\zeta, t')|^2 \langle \rho(t - t') \rangle dt' \ , \end{aligned} \tag{7}$$

where

$$\langle \rho(t) \rangle = \frac{1}{T(t)} \int_{T(t)} \rho(t') dt' = \sum_i \frac{a_i^2}{T_i} \int_{T_i} \delta(t' - t_i) dt \ . \tag{8}$$

We now choose the interval T_i so that it is of length $T_i = (t_{i+1} - t_{i-1})/2$, i.e., corresponding to a time integration over the range $(t_i + t_{i-1})/2$ to $(t_{i+1} + t_i)/2$. This means that the interval contains a single δ-distribution corresponding to pulse i and therefore $\langle \rho(t - t') \rangle$ is piecewise constant with magnitude $\frac{1}{T_i}$ in each interval between δ-distributions with no gaps. Writing

$$T_i = \frac{t_{i+1} - t_{i-1}}{2} = \frac{s^{-1}(z_{i+1}) - s^{-1}(z_{i-1})}{2} \ , \tag{9}$$

and noting that $s^{-1}(z) = t$ such that $ds^{-1}/dz = 1/V(z)$, where now the velocity function is expressed as a function of z rather than t, we may use the Taylor expansion $s^{-1}(z_{i\pm1}) = s^{-1}(z_i) \pm \Delta z ds^{-1}/dz + \ldots$ to yield

$$T_i = \frac{\Delta z}{V_i} + \mathcal{O}(\Delta z^3 d^2/dz^2(1/V)|_i) \, , \tag{10}$$

where $V_i = V(z_i)$. Taking only the leading order term under the assumption that $\Delta z^2 V d^2(1/V)/dz^2|_i \ll 1$ and higher terms are smaller, we obtain

$$\langle\rho(t)\rangle = \sum_i a_i^2 \frac{V_i}{\Delta z} \mathrm{rect}\left[\frac{V_i(t-t_i)}{\Delta z}\right] \, , \tag{11}$$

which is the aforementioned piecewise constant with magnitude $\frac{1}{T_i}$. Hence, if an effective constant "density" $\langle\rho(t)\rangle$ is required, then the relative amplitude of each pulse needs to be modulated by $a_i^2 \propto \Delta z/V_i$. However, if $a_i^2 = \Delta z/V_i$ then $\langle\rho(t)\rangle = 1$ and therefore the peak intensity is at $\zeta = 0$;

$$U_0 = \langle U_P(0,t)\rangle = \int |g(0,t')|^2 dt' \, , \tag{12}$$

Hence, the amplitude is required to be

$$a_i^2 = U_0 \frac{\Delta z}{V_i \int |g(0,t')|^2 dt'} \, . \tag{13}$$

Finally, for N_θ pulse trains crossing at the axis, we should divide the amplitude of each pulse by N_θ to achieve a ponderomotive potential of U_0. Hence, for a Gaussian pulse of the form $g(z,t) = \exp\left[-2z^2/w_0^2 - 2t^2/\tau_0^2\right]$,

$$a_i^2 = U_0 \sqrt{\frac{2}{\pi}} \frac{\Delta z}{N_\theta V_i \tau_0} \, . \tag{14}$$

Therefore, for a (time averaged) intensity profile of the form $I = |I_0 h(\zeta)|$, where $h(\zeta) \in [0,1]$ describes the effective pulse shape, with $\zeta = z - s(t)$ with $s(t) = \int_0^t V(t')dt'$, such that the effective pulse is traveling at group velocity V, then we need to combine a train of transverse pulses described by the pulse shape

$$I_i = I_0 \sqrt{\frac{2}{\pi}} \frac{\Delta z}{N_\theta V_i \tau_0} |g(z-z_i, t-t_i)|^2 \, , \tag{15}$$

with $|g(z-z_i,0)|^2 = h(\zeta)$, $z_i = i\Delta z$ and $t_i = s^{-1}(z_i)$, with $s^{-1}(z) = \int_0^z dz'/V(z')$ and where I_i is the intensity of individual pulses in the train. The time averaging and weighting with a_i^2 to achieve constant time averaged intensity for a series of Gaussian pulses with waist $w = 4$ and duration $\tau = 16$ is shown in Supplementary Fig. 1.

II. MONOENERGETIC BEAM PRODUCTION AND LOCALIZED INJECTION

Using a similar accelerating structure to that discussed in the text, monoenergetic electron beams can be produced using TPA. By causing injection of the electron beam at the start of the plasma column and accelerating over 1525 µm, an 800 MeV electron beam was produced with a 1.9% energy spread, resulting in an acceleration gradient of 0.52 TeV/m. The emittance of the beam was calculated to be 1.38 mm-mrad. The spectrum of this beam is shown in the left panel of Supplementary Fig. 3.

Using the scalings in [1], the dephasing length of a traditional laser wakefield accelerator operating at the same density of 0.012 n_{cr} can be calculated to be 360 µm. This calculation assumes matched conditions with a beam waist of 50 µm and a laser pulse with the same total energy that it took to produce our accelerating structure (16.4 J) in this specific example.

Producing a monoenergetic beam requires localized injection at the start of the plasma. Supplementary Fig. 2 shows plots of a time series of the injection event and wake formation. Notice that the electron charge gets pushed away from the center of the plasma column by the laser beamlets, until the attractive force from the ion channel that gets left behind overcomes the ponderomotive push of the beamlet arrays. Then, the electrons in the plasma collapse back into the plasma column, which produces a wake and injects charge into the accelerating structure at a specific location and time of the simulation. This structure is then continually sustained by additional incoming laser

beamlets until a desired beam energy is produced.

By tracking the particles in the monoenergetic beam throughout the simulation, we can plot the initial positions of the particles in the beam. The right panel of Supplementary Fig. 3 illustrates the localized injection within 5 µm of the start of the plasma column. This localized injection is a result of the sudden collapse of the plasma column back onto itself after the initial perturbation by the beamlet arrays.

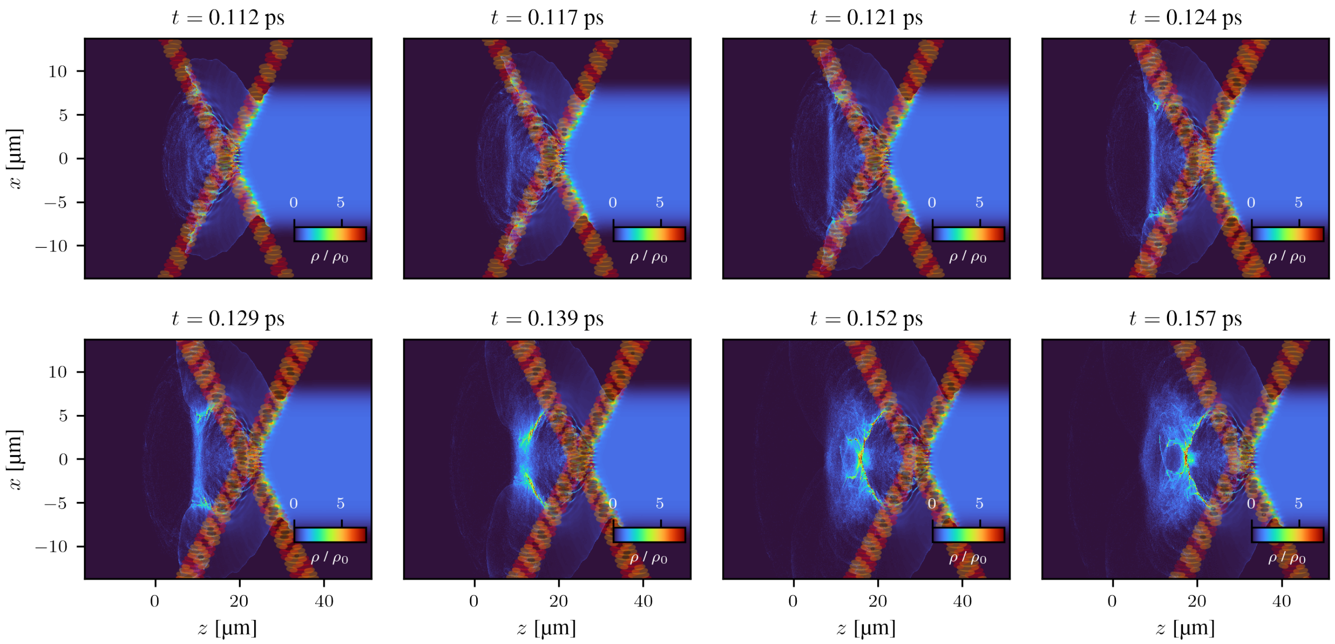

Supplementary Figure 2. Time evolution of the wake formation and injection dynamics. Earliest time is in the top left, latest time is in the bottom right.

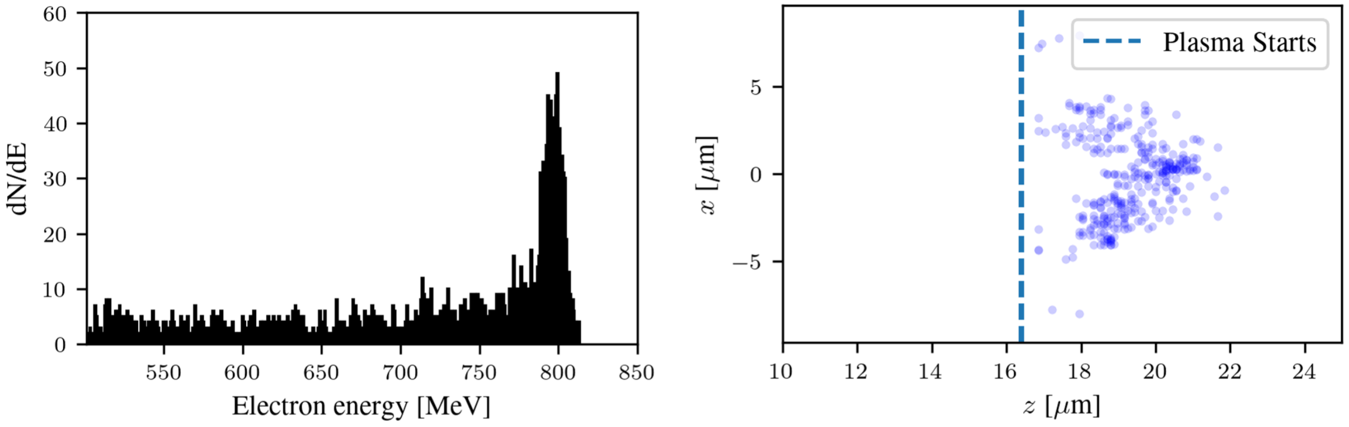

Supplementary Figure 3. (Left) Electron spectrum of monoenergetic beam. The energy spread is 1.9%. (Right) Initial positions of the electrons in the 800 MeV monoenergetic electron beam. Note that the particles are injected within 5 µm of the start of the plasma.

SUPPLEMENTARY REFERENCES

[1] W. Lu, M. Tzoufras, C. Joshi, F. S. Tsung, W. B. Mori, J. Vieira, R. A. Fonseca, and L. O. Silva, Generating multi-gev electron bunches using single stage laser wakefield acceleration in a 3d nonlinear regime, Phys. Rev. ST Accel. Beams **10**, 061301 (2007).
