# Supplementary material for: Transversely pumped laser driven particle accelerator
Source: Nat Commun. 2026 May 2;17:5949. doi: 10.1038/s41467-026-72697-x (PMC13341759; doi:10.1038/s41467-026-72697-x)
Supplement: Supplementary file 2 — Transparent Peer Review file [file 41467_2026_72697_MOESM2_ESM.pdf]

nature portfolio

Peer Review File

Transversely pumped laser driven particle accelerator

Corresponding Author: Mr Tanner Nutting

This manuscript has been previously reviewed at another journal. This document only contains information relating to versions considered at Nature Communications.

This file contains all reviewer reports in order by version, followed by all author rebuttals in order by version.

Attachments originally included by the reviewers as part of their assessment can be found at the end of this file.

Version 0:

Reviewer comments:

Reviewer #2

(Remarks to the Author)
The idea of using multiple low-energy laser pulses to drive a wakefield is extremely interesting, as it offers a wide range of wakefield parameters, including the possibility of controlling its velocity. A second advantage is that it requires lower-energy laser beams, which is particularly important for applications that demand high repetition rates. The challenge lies in translating this idea into practical particle beam parameters, as numerous technical difficulties may arise that could reduce its impact. Nevertheless, the improvements made in previous evaluation rounds, together with the elegance of the idea, make the paper competitive for consideration in Nature Communications.

I suggest that the authors add Cazairgues et al., Nature Photonics, 14, 457–479 (2020), which was submitted on February 18, 2019, well in advance of Ref. [8], submitted on October 24, 2019. The following sentence should also be revised to properly reflect the contributions of the different teams and to correct a typo:
“Palastro et al. [8] pioneered the use of the flying focus concept in LWFA, where the intensity peak of a focused, radially delayed laser pulse travels at a controllable velocity by means of a stepped echelon and an axiparbaola [11–13].”
should be replaced with:
“Cazairgues et al. [*] and Palastro et al. [8] pioneered the use of the luminal wakefield and flying focus concepts in LWFA, where the intensity peak of a focused, radially delayed laser pulse travels at a controllable velocity by means of a stepped echelon and an axiparabola [11–13].”
Additional typos and stylistic suggestions:
• Original:
“This may effect beam properties such as emittance and divergence and could be studied more closely in future work.”
Corrected:
“This may affect beam properties such as emittance and divergence and could be studied more closely in future work.”
• Regarding the expression “a charge of negative –78 pC”:
It is clearer to simply state:
“The electron beam charge is 78 pC.”
(Readers will already understand that electrons carry a negative charge.)
Line 418 (please confirm placement in the manuscript):
Original:
“The second three-dimensional simulation is presented in this work to show that the accelerating structure needs not to occupy the entire width of the plasma column.”
Improved:
“The second three-dimensional simulation presented in this work demonstrates that the accelerating structure does not need to occupy the entire width of the plasma column.”
Comment on discussion clarity:
I find the following discussion unclear. While I agree that the wakefield, being non-cylindrically symmetric, does not extend across the full plasma width, a filament is nevertheless produced. Furthermore, the transverse sizes are so small that I do not see a practical advantage—or relevance—in using a macroscopic plasma. A gas cell would not be transparent, and a gas jet will always be larger than the typical transverse sizes mentioned.

Electron acceleration:
Please clarify how electrons are injected. Are they self-injected? Transversely injected?
Plasma ionization and refraction:
The simulations are performed with pre-ionized plasma. What about refraction effects induced by ionization? How would a large-aperture, high-intensity laser pulse (a_0 = 2.5, waist = 1.94 μm) propagate through hundreds of micrometers of gas before colliding with the second laser pulse?
Experimental feasibility:
Could you provide a schematic illustrating the feasibility of the experimental setup, particularly regarding the placement of multiple large-aperture (f/2) optics within a confined space?

Reviewer #3

(Remarks to the Author)
Comments are submitted as attachment.

Version 1:

Reviewer comments:

Reviewer #2

(Remarks to the Author)
I appreciate the exchanges with the authors, the quality of their responses and the changes they made accordingly.

The new version of the manuscrit is reaching now all the criteria for being published in Nature Communications.

Open Access This Peer Review File is licensed under a Creative Commons Attribution 4.0 International License, which permits use, sharing, adaptation, distribution and reproduction in any medium or format, as long as you give appropriate credit to the original author(s) and the source, provide a link to the Creative Commons license, and indicate if changes were made.
In cases where reviewers are anonymous, credit should be given to 'Anonymous Referee' and the source.
The images or other third party material in this Peer Review File are included in the article's Creative Commons license, unless indicated otherwise in a credit line to the material. If material is not included in the article's Creative Commons license and your intended use is not permitted by statutory regulation or exceeds the permitted use, you will need to obtain permission directly from the copyright holder.
To view a copy of this license, visit https://creativecommons.org/licenses/by/4.0/

Dear Reviewers,

We thank you for the careful time and attention given to our manuscript. Your feedback led to substantial improvements in both clarity and substance, and we believe the revised manuscript now presents a more compelling and better-grounded case for Transversely Pumped Acceleration (TPA). In particular, we have strengthened the paper's practical outlook by adding a unified feasibility discussion and a clear path toward an experimentally impactful proof-of-principle demonstration, addressing the central concerns raised about implementation, scalability, and realistic experimental geometry.

Below, we provide a point-by-point response to each reviewer comment. Reviewer comments are reproduced in red text, followed by our responses and a description of the corresponding changes in the manuscript, including line numbers in the revised version. Modifications to the updated manuscript are highlighted in green.

In summary, this work introduces Transversely Pumped Acceleration (TPA), a new class of laser-driven plasma accelerator in which arrays of transversely propagating, time-delayed laser beamlets drive an accelerating structure whose effective phase velocity can be tuned by design. This approach provides a route to sustained acceleration beyond the conventional dephasing and depletion constraints that limit longitudinal wakefield schemes, while enabling new capabilities such as programmable wake velocity, the separation of injection from acceleration, very high electron/ion energies, and very high repetition rates.

A key advance in the revision is that we now articulate a realistic experimental architecture and staged validation program. We show that TPA does not require an impractical number of independently aligned fast optics near the target. The essential requirement is generation of a moving region of beam overlap along a shared focal line, which can be achieved using beamlet arrays combined with a small number of large-aperture cylindrical focusing optics. This geometry relaxes alignment constraints via astigmatic line focusing and shifts the dominant engineering challenge from optical crowding to single-aperture energy transport and fluence management, an approach aligned with established large-aperture laser engineering practice. We further outline a three-step proof-of-principle roadmap (wake excitation, velocity control, and scaling) to demonstrate feasibility in a controlled progression before pursuing aggressive parameter sets.

Together, these revisions clarify both the conceptual novelty and the practical trajectory of TPA, including its potential relevance to high-repetition-rate operation and scalable architectures. We believe the revised manuscript is now suitable for further consideration in *Nature Communications*.

REVIEWER COMMENTS

Reviewer #2 (Remarks to the Author):

Reviewer Comment:
The idea of using multiple low-energy laser pulses to drive a wakefield is extremely interesting, as it offers a wide range of wakefield parameters, including the possibility of controlling its velocity. A second advantage is that it requires lower-energy laser beams, which is particularly important for applications that demand high repetition rates. The challenge lies in translating this idea into practical particle beam parameters, as numerous technical difficulties may arise that could reduce its impact. Nevertheless, the improvements made in previous evaluation rounds, together with the elegance of the idea, make the paper competitive for consideration in Nature Communications.

Author Response:
We thank the reviewer for their time spent helping us improve the manuscript. The idea proposed in our manuscript allows for significant advantages in the field of laser driven particle acceleration. To address concerns regarding practicality we have added a discussion in the conclusion of the manuscript s**ee lines 563 to 659**.

Reviewer Comment:
I suggest that the authors add Cazairgues et al., Nature Photonics, 14, 457–479 (2020), which was submitted on February 18, 2019, well in advance of Ref. [8], submitted on October 24, 2019. The following sentence should also be revised to properly reflect the contributions of the different teams and to correct a typo:
“Palastro et al. [8] pioneered the use of the flying focus concept in LWFA, where the intensity peak of a focused, radially delayed laser pulse travels at a controllable velocity by means of a stepped echelon and an axiparbaola [11–13].”
should be replaced with:
“Cazairgues et al. [*] and Palastro et al. [8] pioneered the use of the luminal wakefield and flying focus concepts in LWFA, where the intensity peak of a focused, radially delayed laser pulse travels at a controllable velocity by means of a stepped echelon and an axiparabola [11–13].”

Author Response:
The changes have been implemented to properly reflect various contributions to the ideas discussed in the introduction. **See lines 46 to 51.**

Reviewer Comment:
Additional typos and stylistic suggestions:
• Original:
“This may effect beam properties such as emittance and divergence and could be studied more closely in future work.”
Corrected:

"This may affect beam properties such as emittance and divergence and could be studied more closely in future work."
• Regarding the expression "a charge of negative –78 pC":
It is clearer to simply state:
"The electron beam charge is 78 pC."
(Readers will already understand that electrons carry a negative charge.)
Line 418 (please confirm placement in the manuscript):
Original:
"The second three-dimensional simulation is presented in this work to show that the accelerating structure needs not to occupy the entire width of the plasma column."
Improved:
"The second three-dimensional simulation presented in this work demonstrates that the accelerating structure does not need to occupy the entire width of the plasma column."

Author Response:
We thank the reviewer for pointing out the typos and we agree with the stylistic changes. These changes have been implemented in **lines 273, 405, 406, and 425-428.**

Reviewer Comment:
Comment on discussion clarity:
I find the following discussion unclear. While I agree that the wakefield, being non-cylindrically symmetric, does not extend across the full plasma width, a filament is nevertheless produced. Furthermore, the transverse sizes are so small that I do not see a practical advantage—or relevance—in using a macroscopic plasma. A gas cell would not be transparent, and a gas jet will always be larger than the typical transverse sizes mentioned.

Author Response:
We thank the reviewer for bringing this lack of clarity to our attention. We have removed the discussion regarding the macroscopic plasma and have simplified the discussion around the plasma filament. **See line 433** where the following text has been removed to help the flow of the manuscript: "This is an advantage with regards to the practical implementation of the concept, as this shows that a plasma filament is not necessary to produce an accelerating structure. This result suggests that the accelerating structure could be produced in a macroscopic plasma."

Reviewer Comment:
Electron acceleration:
Please clarify how electrons are injected. Are they self-injected? Transversely injected?

Author Response:
In the simulations demonstrated in the text, the electrons are injected via self injection from the collapse of the plasma column back into itself in the wake of the crossing laser pulses. In practice, ionization injection, shock injection, or even injection using a separate linac could be used. This has been added to the text in **lines 362 to 366.**

Reviewer Comment:
Plasma ionization and refraction:
The simulations are performed with pre-ionized plasma. What about refraction effects induced by ionization? How would a large-aperture, high-intensity laser pulse (a_0 = 2.5, waist = 1.94 μm) propagate through hundreds of micrometers of gas before colliding with the second laser pulse?

Author Response:
This is an important point to discuss and we thank the reviewer for opening the discussion. Using a plasma filament would reduce these refractive effects. Instead of propagating through hundreds of microns of plasma, the beam would only propagate through tens of microns of plasma. Any refractive effects could be accounted for prior to the beams entering the plasma since the discrete nature of the idea allows for such fine tuning. It is also possible to preionize the plasma. This discussion has been added to the text in **lines 434 to 440.**

Reviewer Comment:
Experimental feasibility:
Could you provide a schematic illustrating the feasibility of the experimental setup, particularly regarding the placement of multiple large-aperture (f/2) optics within a confined space?

Author Response:
We have added a discussion regarding the feasibility of a real experiment and have proposed a realistic layout of the scheme using beamlets and two cylindrical focusing optics. In this section, we have added a schematic of the idea which replaces the old Figure 6. **See lines 483 to 490 and 564 to 659** for the discussion.

<u>Reviewer #3 (Remarks to the Author):</u>

Reviewer Comment:
I have reviewed the author's response to all reviewers' comments and the revised manuscript. While I appreciate the effort made to address the concerns, I believe the major weaknesses of the initial manuscript continue to be present. Much of the response from the authors is framed around potential workarounds for individual challenges, rather than incorporating the solutions into a unified vision for conducting an impactful experiment. In my view, the ideas presented in the manuscript (while possessing significant potential) remain too underdeveloped for the high-impact venue the authors are targeting.

Author Response:
We thank the reviewer for the insightful comments and we have improved our manuscript as a result. Understanding the importance of a path towards experimental demonstration, we have added an extended discussion in the manuscript in **lines 564 to 659** to address practical concerns.

Reviewer Comment:
The issue of feasibility

The authors present two responses to the criticism regarding feasibility. First, they propose that a proof of principle experiment would be reasonably simple to implement. Second, they suggest that feedback control and machine learning techniques could be deployed to address the technically challenging aspects of the scheme, such as synchronizing multiple pulses with sub-femtosecond precision and focusing them to 3 μm with micron-level accuracy. Both capabilities remain state-of-the art research problems for less demanding conditions than would be required for the proposed method. My views on the feasibility of these methods is much closer to reviewer #2 than those of the authors: synchronizing and aligning lasers with much more relaxed tolerances (>10 microns and >10 femtosecond jitter) remain a significant challenge for LWFA experiments even with a small number of laser pulses at moderate energies and it is not at all obvious that the order of magnitude improvement the authors imagine are feasible for tens to thousands of pulses required for this scheme to work over appreciable distances.

Author Response:
We thank the reviewer for helping us improve the manuscript by directly addressing these feasibility concerns. As discussed in the new section, fiber arrays could be used in tandem with cylindrical focusing optics. The use of a cylindrical focusing optic dramatically reduces the alignment tolerances since the beamlets can be overlapped from many astigmatic foci. The temporal jitter tolerances are well within the range of what has been achieved in coherent fiber combining [23], where DPAMs have been used to control the phase, and therefore timing of pulses to lambda/90 RMS error. This corresponds to sub-fs jitter for 1 μm laser pulses. This is now addressed in the manuscript in **lines 564 to 601**.

Reviewer Comment:
The perceived challenges in feasibility also undermine the claim of scalability, which the authors identify as one of the principal advantages of the scheme. This is in the sense that each additional laser beamlet introduces increasing complexity, with difficulty often increasing nonlinearly with the number of beamlets introduced. This is because each beamlet must be timed and aligned with high precision relative to the preceding and the following beamlets in order to maintain the desired focus velocity.

Author Response:
The newly proposed implementation could use fiber arrays in the near field, which could be added on to the apparatus more simply than aligning free propagating beams. Each beamlet in a given beamlet array would share a single focusing optic (a cylindrical mirror), reducing the alignment tolerances from the overlapped foci and reducing the complexity of aligning the system by reducing the total number of focusing optics to be aligned. This is now addressed in **lines 564 to 601**.

Reviewer Comment:
I would also reiterate a point from my initial review: a proof-of-principle experiment is best way to demonstrate feasibility and would significantly strengthen the case for publication. This does not require experiments in a facility on the scale of ZEUS; even 4–10

pulses at the 7 mJ level would be sufficient to make the case.

Author Response:
We recognize the importance of a proof of principle experiment. For these reasons, we have added an outline to an experimental demonstration to the manuscript.

- Proof-of-principle wake excitation: Demonstrate that two opposing arrays of a modest number of beamlets, line-focused with cylindrical optics, can drive a wake whose excitation follows the programmed delay pattern.
- Velocity control: Vary the imposed beamlet delays and directly observe the resulting change in the effective wake velocity.
- Scaling: Increase beamlet number and reduce spot size only after the control principle has been experimentally validated.

This approach emphasizes that feasibility is established not by immediately realizing the most aggressive parameter set, but by experimentally verifying the core control mechanism on which scalability rests. It builds up the framework of the experiment in a controlled way.

Proof of principle experiments could be conducted with apodized beams (such as from ZEUS) and a pulse front tilt.

This has been added to the text in **lines 621 to 638**.

Reviewer Comment:
The issues related to electron acceleration application
As I said in the original review, this scheme will have difficulty competing with the currently well-established LWFA methods. The mitigation of dephasing, which the authors identify as the other principal advantage of this scheme is undermined by the properties of the beam produced by such structure, at least as presented. Aside from the asymmetry of the structure, for which the authors present a plausible, but untested solution, the simulated beam quality (while reasonable) does not advance any critical performance parameter. The produced beam has ~800 MeV, sub-pC charge, ~ 200 A current, ~1 micron emittance, and 2% energy spread. Standard LWFA experiments today routinely generate 1–10 GeV beams with greater charge and comparable or better quality. That is not to mention the additional challenges posed by the asymmetry of the structure, which would become relevant in full 3D simulations and realistic implementation of this method.

Author Response:
We appreciate the comment. However, we disagree with the main premise of the comment: that TPA does not advance the performance of laser driven electron acceleration. With our newly devised plan for experimental implementation, we show that scaling the accelerator is simpler than initially perceived. It is the scalability of this acceleration scheme that sets it apart from any other dephasingless concepts. We have further emphasized this point in the discussion section

in **lines 511 to 526**. Note also that we have run full 3D simulations and did not observe any negative consequences in terms of acceleration as a result of the asymmetric beam.

Reviewer Comment:
Moreover, extending the acceleration length beyond the dephasing limit is not a key concern for 1–10 GeV beams, where the required lengths remain manageable (on the order of 1–20 cm). It is however an important consideration where hundreds of GeV of energies are sought — mainly in collider applications — and for that regime, beam quality must improve substantially in every dimension beyond what is demonstrated here.

Author Response:
Thank you for the comment. We acknowledge the beam quality is not perfect in this specific example. An advantage of the scheme is that the use of low power lasers allows for high repetition rate corrections to beam parameters by adjusting laser beamlet parameters and timings. Beam quality could be tailored to a given application dynamically based on figures of merit set by the user. We have added this discussion to the text in the context of improving beam quality in **lines 520 to 526**.

Reviewer Comment:
In summary, while the manuscript presents novel ideas with significant potential, the concerns raised by myself and other referees highlight important questions about the current level of technical feasibility and likely payoff, which will need to be addressed before the work can be considered further.

Author Response:
We thank you for reviewing our manuscript. We have addressed the reviewer concerns and have devised a feasible plan for implementation of the idea in a proof of principle experiment.

I have reviewed the author's response to all reviewers' comments and the revised manuscript. While I appreciate the effort made to address the concerns, I believe the major weaknesses of the initial manuscript continue to be present. Much of the response from the authors is framed around potential workarounds for individual challenges, rather than incorporating the solutions into a unified vision for conducting an impactful experiment. In my view, the ideas presented in the manuscript (while possessing significant potential) remain too underdeveloped for the high-impact venue the authors are targeting.

The issue of feasibility

The authors present two responses to the criticism regarding feasibility. First, they propose that a proof of principle experiment would be reasonably simple to implement. Second, they suggest that feedback control and machine learning techniques could be deployed to address the technically challenging aspects of the scheme, such as synchronizing multiple pulses with sub-femtosecond precision and focusing them to 3 μm with micron-level accuracy. Both capabilities remain state-of-the art research problems for less demanding conditions than would be required for the proposed method. My views on the feasibility of these methods is much closer to reviewer #2 than those of the authors: synchronizing and aligning lasers with much more relaxed tolerances (>10 microns and >10 femtosecond jitter) remain a significant challenge for LWFA experiments even with a small number of laser pulses at moderate energies and it is not at all obvious that the order of magnitude improvement the authors imagine are feasible for tens to thousands of pulses required for this scheme to work over appreciable distances.

The perceived challenges in feasibility also undermine the claim of scalability, which the authors identify as one of the principal advantages of the scheme. This is in the sense that each additional laser beamlet introduces increasing complexity, with difficulty often increasing nonlinearly with the number of beamlets introduced. This is because each beamlet must be timed and aligned with high precision relative to the preceding *and* the following beamlets in order to maintain the desired focus velocity.

I would also reiterate a point from my initial review: a proof-of-principle experiment is best way to demonstrate feasibility and would significantly strengthen the case for publication. This does not require experiments in a facility on the scale of ZEUS; even 4–10 pulses at the 7 mJ level would be sufficient to make the case.

The issues related to electron acceleration application

As I said in the original review, this scheme will have difficulty competing with the currently well-established LWFA methods. The mitigation of dephasing, which the authors identify as the other principal advantage of this scheme is undermined by the properties of the beam produced by such structure, at least as presented. Aside from the asymmetry of the

structure, for which the authors present a plausible, but untested solution, the simulated beam quality (while reasonable) does not advance any critical performance parameter. The produced beam has ~800 MeV, sub-pC charge, ~ 200 A current, ~1 micron emittance, and 2% energy spread. Standard LWFA experiments today routinely generate 1–10 GeV beams with greater charge and comparable or better quality. That is not to mention the additional challenges posed by the asymmetry of the structure, which would become relevant in full 3D simulations and realistic implementation of this method.

Moreover, extending the acceleration length beyond the dephasing limit is not a key concern for 1–10 GeV beams, where the required lengths remain manageable (on the order of 1–20 cm). It is however an important consideration where hundreds of GeV of energies are sought — mainly in collider applications — and for that regime, beam quality must improve substantially in every dimension beyond what is demonstrated here.

In summary, while the manuscript presents novel ideas with significant potential, the concerns raised by myself and other referees highlight important questions about the current level of technical feasibility and likely payoff, which will need to be addressed before the work can be considered further.
